# Effects of mixed carriers on diatomite supported nano-TiO_2_

**DOI:** 10.1038/s41598-022-24441-w

**Published:** 2022-11-18

**Authors:** Hailan Jin, Honglei Chen, Xueren Qian, Wanqi Jin, Xinyue Du, Can Jin, Haojun Li, Hao Li, Yumo Zhu, Junhyung Chao

**Affiliations:** 1 State Key Laboratory of Biobased Material and Green Papermaking, Qilu University of Technology, Shandong Academy of Sciences, Jinan, 250353 China; 2 Key Laboratory of Bio-based Material Science & Technology (Northeast Forestry University) Ministry of Education, No. 26 Hexing Road, Harbin, 150040 China; 3 College of Material Science and Engineering, Northeast Forestry University, Harbin, 150040 China; 4 Department of Paper Science, College of Forestry Environmental Sciences, Kangwon National University, Chunchon-si, 200701 Korea

**Keywords:** Materials science, Nanoscience and technology

## Abstract

In this study, calcium carbonate, sepiolite, and commonly used diatomite (DE) carriers were mixed to prepare calcium carbonate or sepiolite mixed DE/nano-titanium dioxide (TiO_2_). The analyses of X-ray diffraction and scanning electron microscope confirmed that the particle size of nano-TiO_2_ was about 20–24 nm in DE/nano-TiO_2_, and the particles were relatively uniform. When (calcium carbonate and sepiolite + DE)/nano-TiO_2_ was used, the Ti content in the composite remained unchanged, while the particle size of nano-TiO_2_ increased to a certain extent. Among them, the use of (calcium carbonate + DE)/nano-TiO_2_ increased the Ti content in the composite material significantly. Therefore, the findings demonstrated the feasibility of nano-TiO_2_ supported by the mixed carrier.

## Introduction

Air and water pollution control are processes of protecting the environment from environmental pollutants, reducing or eliminating the production of by-products that are harmful to humans and the environment. Recently, improved photocatalytic technologies have been rapidly developed. Titanium dioxide (TiO_2_) photocatalytic oxidation technology—an efficient air treatment technology—is characterized by strong oxidation and a mild reaction with no secondary pollution. This technology has been widely used to produce high-efficiency photocatalysts^1^. However, these catalysts are easily recycled when applied to treat pollutants. Legrini et al.^2^ proposed that the curing of TiO_2_ and the development of new photoreactors are the used nano-TiO_2_ supported on some carriers, which is an effective way to solve its practical application problems. The carriers used for nano-TiO_2_ curing are mostly inorganic materials, such as porous silica gel, ceramics, glass, and activated carbon^3–5^.

Diatomaceous earth (DE) is a biogenic siliceous sedimentary rock mainly composed of ancient diatom remains. High-purity DE has a three-dimensional pore structure and good adsorption performance. The main component of DE is amorphous hydrous silica (more than 80%), which is a chemically stable and eco-friendly material^6,7^. DE is a rare inorganic carrier material with good light transmittance and excellent absorbability. These excellent properties are due to the structural uniqueness of DE nanopores, which significantly enhances the adsorption of reactants and improves the performance of the active components of catalysts. The main functions of DE/TiO_2_ composite photocatalyst are to prevent the loss of TiO_2_ powder particles for easy recycling and to overcome the shortcomings of suspended phase TiO_2_. The TiO_2_ is supported on the surface of the carrier to increase its specific surface area and improve its utilization rate^8^. Recently, several studies have been conducted on DE-supported TiO_2_ photocatalysts, and the findings of these studies have proven that DE is an excellent TiO_2_ carrier^9,10^. Supporting nano-TiO_2_ on DE can enhance the adsorption performance of photocatalytic composites and improve the photocatalytic performance of nano-TiO_2_^11^. Zhang et al.^12^ used titanium sulfate as a precursor to prepare a DE/TiO_2_ composite photocatalyst via a simple hydrolysis deposition method. The composite showed higher photocatalytic performance than TiO_2_ in formaldehyde degradation. The cellulose-based DE/TiO_2_ catalytic material with a uniform structure, several micropores, and a large surface area can improve air purification efficiency, leading to its wide application.

Calcium carbonate, a type of white, non-metallic mineral filler, is one of the main components of diatom mud; it is also cheap and readily available in the market^13–16^. Calcium carbonate has high whiteness and good hiding power. The filler (diatom mud) is commonly used for producing papers and interior decoration materials. Hu et al.^17,18^ found that some calcium carbonate penetrated and modified the pores of DE during the calcination process. This modification transformed macropores to mesopores in DE. The prepared mesoporous DE/calcium carbonate performed better than that of DE. Sepiolite is a pure natural hydrated magnesium-rich silicate clay mineral, and it is part of the monoclinic pseudo-orthorhombic group. Sepiolite is characterized by good adsorption, rheology, and catalytic properties owing to its unique crystal structure, leading to its wide application range. The products are mainly used in conventional catalyst carriers, thickener production, and mud drilling, and also as suspending agents for papermaking and additives for building materials or chemicals^19^. Due to the synergistic effect between the strong adsorption capacity of sepiolite and the high catalytic activity in TiO_2_, the TiO_2_/sepiolite composite exhibits excellent performance in photocatalytic degradation of non-biodegradable organic matter. Zhou et al.^20^ showed that the anatase_–_rutile phase transition only occurred in rare earth (RE)–doped TiO_2_/Sep nanocomposites, while the Ti–O–RE bond that only occurred in the samples could effectively inhibit the aggregation and crystallite growth of TiO_2_. Wang et al.^21^ prepared TiO_2_-supported sepiolite catalytic materials via a gel method and observed that the TiO_2_ penetrated the surface and pores of sepiolite, improving its photocatalytic efficiency.

Therefore, in this study, calcium carbonate and sepiolite were mixed with commonly used DE carriers to prepare calcium carbonate and sepiolite mixed DE/nano-TiO_2_ to investigate the feasibility of the mixed carrier, the growth of nano-titania mixed carrier, and its effect on particle size.

## Material and methods

### Materials

The DE used is a product of Japan Showa Chemical Co., Ltd., and its composition is shown in Table 1. Titanium sulfate and urea were used as precipitants, and DE was used as a TiO_2_ carrier.

**Table 1:** Diatomite chemical components.

Chemical composition	Contents (%)
SiO_2_	79.6
Al_2_O_3_	0.6
Fe_2_O_3_	0.9
CaO	0.3
Igniton loss	18.9

### Sample preparation

Titanyl sulfate and urea solutions (mol/L) were prepared. Quantitative DE powder (DE: TiO_2_ mass ratio = 2:1) was added to the urea solution and stirred evenly. Titanyl sulfate solution was added to the mixture. A concentrated sulfuric acid was added drop-wise to the mixture while stirring to keep the pH value of the solution at 1.5. The mixture was stirred evenly and placed in a water bath, and then heated at 75 °C for 1 h. After completing the reaction, the mixture was cooled, filtered with a vacuum filter, and then washed with distilled water. Barium chloride was added drop-wise to the washing solution until no precipitate was formed in the solution. The obtained product was dried after completing solution washing. The product was calcinated in a muffle furnace at a temperature of 700 °C for 2 h. The titanyl sulfate and urea concentrations were changed to obtain nano-TiO_2_ products with different particle sizes. When preparing the mixed carrier, the dried DE/TiO_2_ was ground. The calcium carbonate and sepiolite of the same quality were added to the DE, then mixed evenly, and finally calcined to obtain a mixed carrier supported nano-TiO_2_ product.

### Calculation of nano-TiO_2_ particle size

For X-ray diffraction (XRD) analysis, a D/max-2200VPC X-ray diffractometer manufactured by Rigaku Corporation was used to determine the crystal form of the sample. The current and voltage were 40 kV and 30 mA, respectively, and the scan rate was 5°/min. The test was performed in the range of 20°–90°.

The grain size is calculated according to the diffraction peak width in the XRD pattern, using the Scherrer formula:

1 $${\text{Dhkl }} = \, \left( {{\text{k}}\lambda } \right)/\left( {\beta {\text{cos}}\theta } \right)$$

where D is average grain size, k is Scherrer constant (0.89), λ is XRD wavelength (0.154 nm), θ is Bragg angle, and β is Integral peak width at half maximum (radians) of the strongest diffraction peak (101).

### Scanning electron microscope (SEM) and X-ray electron spectroscopy (XES) analyses

The samples were observed by Quanta-200 SEM manufactured by FEI Company of America, and the element content was analyzed by a K-Alpha photoelectron spectrometer produced by VG Company.

## Results and discussion

### Preparation of DE/nano-TiO_2_

#### XRD analysis

**Figure 1:**
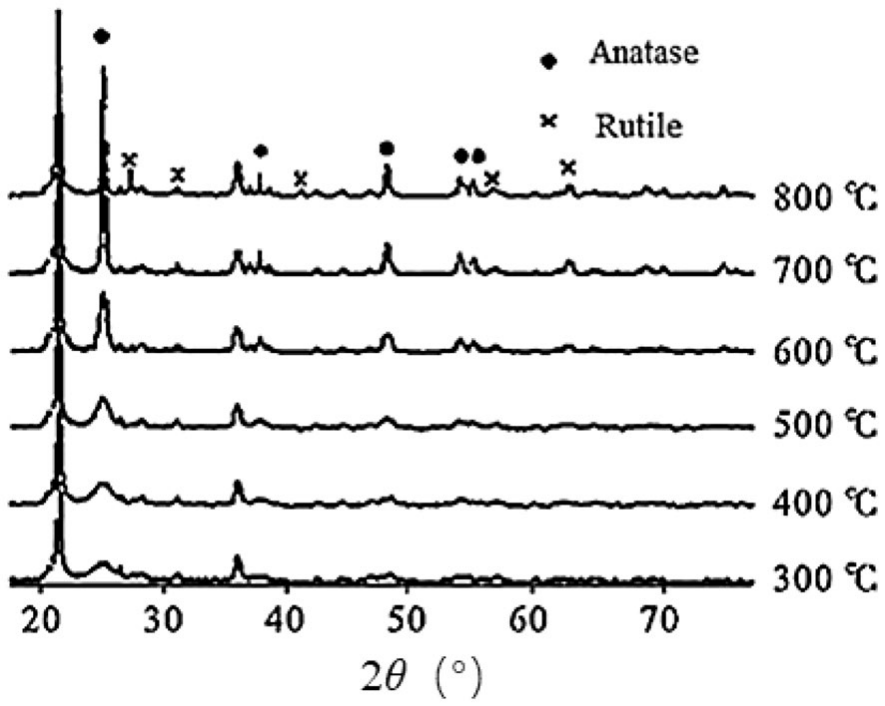
XRD patterns of DE-supported nano-TiO_2_ prepared by precipitation method (TiOSO_4_, Urea) and calcined at 300–800 °C for 2 h^23^.

**Figure 2:**
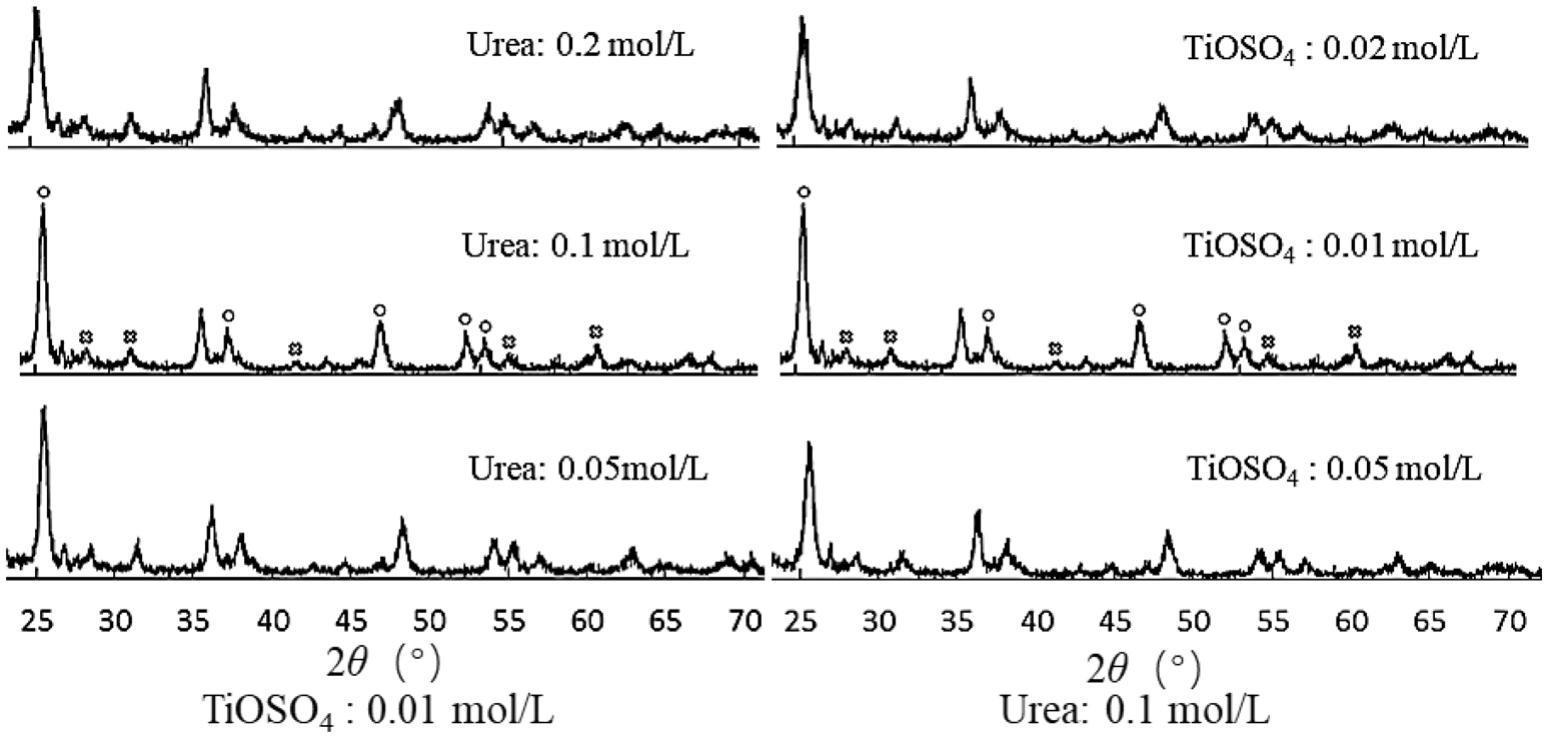
XRD patterns of the prepared DE/nano-TiO_2_ after calcination at 700 °C for 2 h (open circle: Anatase; cross symbol: Rutile).

It is generally believed that the anatase phase in nano-TiO_2_ particles has high photocatalytic activity^22^. The energy gap of anatase nano-TiO_2_ particles (3.2 eV) was slightly larger than that of rutile (3.0 eV). Yu et al.^23^ showed that after the calcination of DE/TiO_2_ at 300–800 °C for 2 h with increasing calcination temperature, the characteristic diffraction peaks of the sharp TiO_2_ phase (2θ = 25.2°) gradually increased, and the crystal form was gradually intact (Fig. 1). When the temperature was increased to 700 °C, the optimal state of the anatase crystal phase appeared. However, when the temperature exceeded 700 °C, the crystal form was significantly affected. The high temperature can also break the nano-titania layer grown on the surface of DE. Wang et al.^24^ also showed that the grain growth was significantly accelerated when the calcination temperature exceeded 650 °C. Thus, a calcination temperature of 700 °C for 2 h was adopted in this experiment. The XRD results are shown in Fig. 2. Figure 2 reveals that the corresponding characteristic peaks appear in the XRD of all samples, which proves that the prepared nano-TiO_2_ is a mixed crystal form of anatase type and rutile type. The characteristic peaks of the anatase phase appeared at 2θ = 37.18° and 47.16°, and the rutile phase appeared at 2θ = 27.14°, 36.10°, and 54.13°.

The particle size of nano-TiO_2_ was calculated using Eq. (1), and the results are shown in Table 2. The particle size of nano-TiO_2_ in all samples was between 20 and 24 nm. When the concentration of titanyl sulfate and urea were 0.01 mol/L and 0.1 mol/L, respectively, the particle size of nano-TiO_2_ was the smallest (20.4 nm). When the urea concentration was fixed, the particle size did not change significantly with the increase of the titanyl sulfate concentration. However, when the concentration of titanyl sulfate was constant with increasing urea concentration, the peak value of the anatase phase and the particle size decreased and increased, respectively. These changes may be attributed to the use of urea as a precipitant, resulting in agglomeration of the particles.

**Table 2:** Particle size of nano-TiO_2_ (nm).

TiOSO_4_ (mol/L)	Urea (mol/L)		
	0.05	0.1	0.2
0.005	21.7	22.8	22.9
0.01	21.3	20.4	21.1
0.02	21.4	22.5	23.5

#### SEM observation

**Figure 3:**
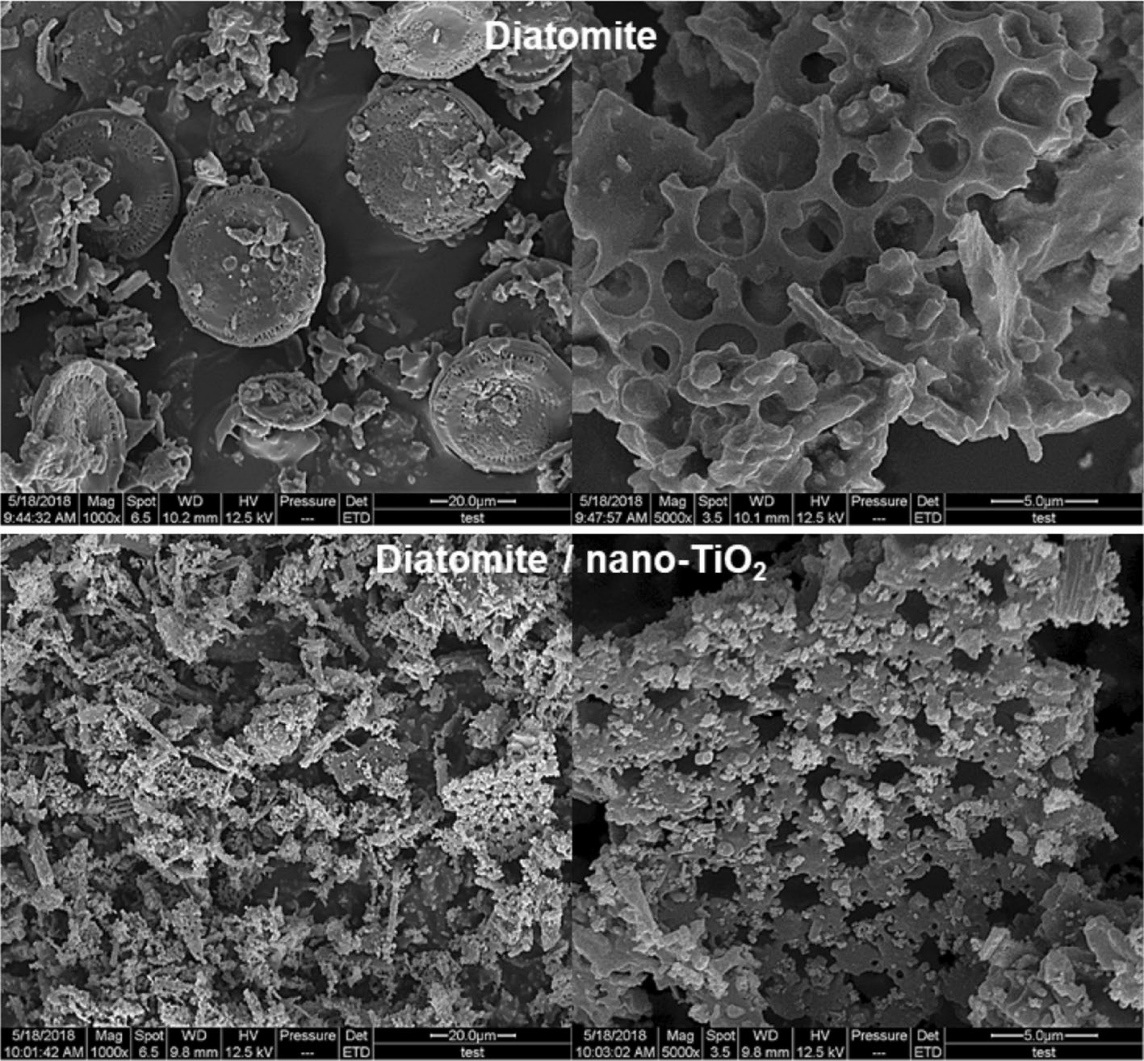
SEM image of DE/nano-TiO_2_.

SEM images of DE and DE/nano-TiO_2_ are shown in Fig. 3. Through the observation of the left SEM image, it is apparent that the nano-TiO_2_ particles were loaded on the surface and voids of the DE, proving that the nano-TiO_2_ was successfully attached. In the right SEM image, the overall damage to DE during loading of nano-TiO_2_ can be observed.

### Effect of mixed carriers on supporting nano-TiO_2_

After confirming that DE successfully loaded on nano-TiO_2_ particles, we studied the effect of mixed carriers on the loading of nano-TiO_2_. From the above experiments, the urea concentration greatly influences the particle size of nano-TiO_2_. In this experiment, we used a 0.01 mol/L concentration of titanyl sulfate to investigate the effect of urea concentration.

The particle size of nano-TiO_2_ was calculated using the XRD detection results of nano-TiO_2_ supported by a mixed carrier, as shown in Table 3. The experimental results show that almost all the mixed carriers tend to increase the particle size of nano-TiO_2_. When the mixture (calcium carbonate + DE) was used as the carrier with 0.1 mol/L urea concentration, the particle size of nano-TiO_2_ became the smallest (33.3 nm). In contrast, when (sepiolite + DE) was used as the carrier, the particle size of nano-TiO_2_ was the smallest, at 15.2 nm when the urea concentration was 0.05 mol/L. However, when the urea concentration was 0.2 mol/L, the particle size of nano-TiO_2_ was the largest, at 56.5 nm. The results showed that the agglomeration of sepiolite was more serious than that of calcium carbonate, which could also be observed in SEM.

**Table 3:** Particle size of nano-TiO_2_ (nm).

Carrier	Urea (mol/L)		
	0.05	0.1	0.2
Diatomite	21.3	20.4	21.1
Diatomite + CaCO_3_	41.8	33.3	38.9
Diatomite + meerschaum	15.2	56.5	24.3

TiOSO_4_: 0.01 mol/L.

**Figure 4:**
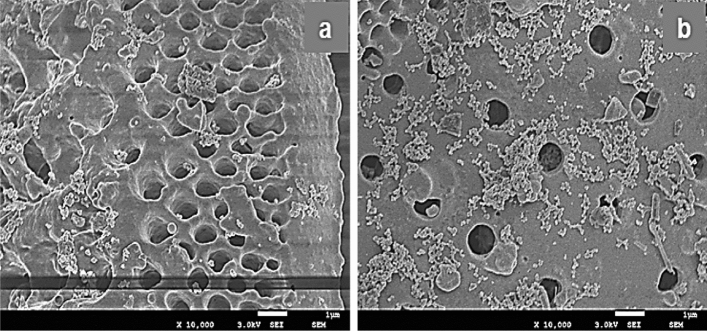
SEM image of DE mixed with calcium carbonate or sepiolite/nano-TiO_2_. (**a**) calcium carbonate + DE + titanyl sulfate 0.01 mol/L + urea 0.1 mol/L; (**b**) sepiolite + DE + titanyl sulfate 0.01 mol/L + urea 0.1 mol/L.

**Figure 5:**
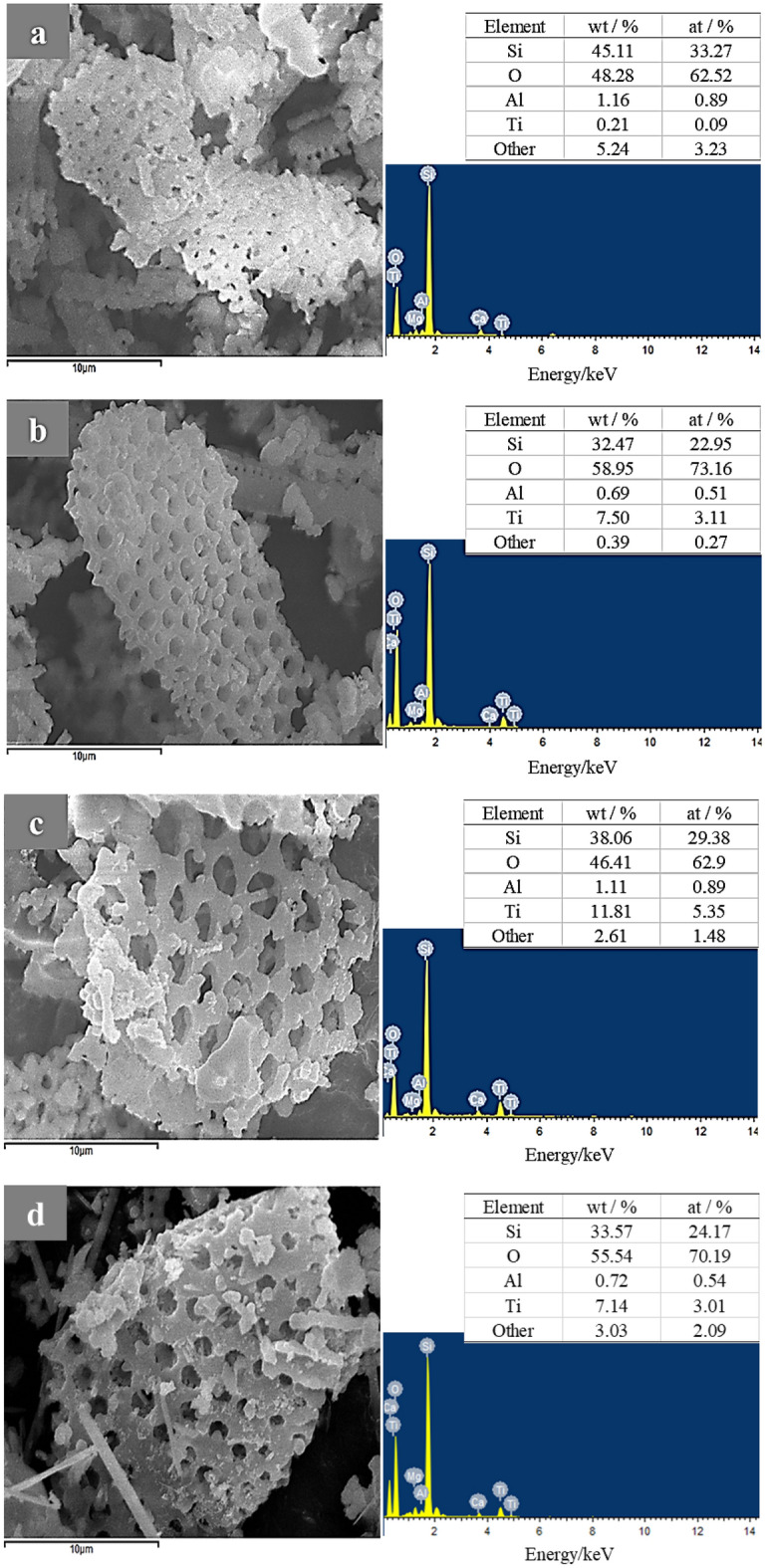
SEM image of the sample and its EDS pattern. (**a**) DE; (**b**) DE/nano-TiO_2_; (**c**) (calcium carbonate + DE)/nano-TiO_2_; (**d**) (sepiolite + diatomite)/nano-TiO_2_.

In the SEM image observation, it was observed that the agglomeration phenomenon of nano-TiO_2_ on the surface of the mixed carrier was increased (Fig. 4). The explosives detection systems (EDS) analysis is shown in Fig. 5a and b revealed that the content of Si and Al in DE/nano-TiO_2_ decreased more than that of meta-DE, whereas the content of Ti increased significantly. When the carrier/nano-TiO_2_ was mixed (Fig. 5c,d), the content of Si and Al decreased to a certain extent, while the content of Ti increased. This variation indicated that the mixed carrier-supported nano-TiO_2_ was feasible. Moreover, in the case of (calcium carbonate + DE)/nano-TiO_2_, the Ti content increased significantly, indicating that the mixture of calcium carbonate improved the growth of nano-TiO_2_ on the surface of DE. This phenomenon can be attributed to the fact that calcium carbonate enters the DE voids during the high temperature of the roasting process, which promotes the transformation of DE from macropores to mesopores^17^. Therefore, this phenomenon will be investigated further in the future.

DE and calcium carbonate are commonly used fillers in papermaking. The DE of the vegetation in this experiment or (calcium carbonate + DE) loaded with nano-TiO_2_ as a composite filler can be used to prepare paper with photocatalytic performance using a papermaking method. The experimental results provide a theoretical basis for paper-based photocatalytic materials.

## Conclusions

The XRD and SEM analyses of the samples confirmed that the particle size of DE/nano-TiO_2_ was approximately 20–24 nm, which were relatively uniform. The particle size of nano-TiO_2_ increased as urea concentration increased. In the case of (calcium carbonate and sepiolite + DE)/nano-TiO_2_, the increase of Ti content in the composite was insignificant. However, in the case of (calcium carbonate + DE)/nano-TiO_2_, the Ti content increased significantly. These findings demonstrated the feasibility of nano-TiO_2_ supported by a mixed carrier.

## Data Availability

The datasets used and/or analyzed during the current study are available from the corresponding author upon reasonable request.
